# An unusual case report of unilateral parotid gland sarcoidosis with spontaneous remission

**DOI:** 10.1097/MD.0000000000018172

**Published:** 2019-12-10

**Authors:** Panagiotis T. Diamantopoulos, Emmanouil Charakopoulos, Nora-Athina Viniou, Lydia Diamantopoulou, Maria Gaggadi

**Affiliations:** a First Department of Internal Medicine, Laikon General Hospital, National and Kapodistrian University of Athens; b Pulmonology Department, Laikon General Hospital, Athens, Greece.

**Keywords:** parotid gland sarcoidosis, granulomatous parotitis, corticosteroids, parotidectomy, sarcoidosis

## Abstract

**Rationale:**

Parotid gland sarcoidosis is a well-recognized, but uncommon disease entity. Parotidectomy is most commonly performed to establish the diagnosis and most patients are treated with corticosteroids.

**Patient concerns:**

A young female patient presented with right parotid enlargement and developed symptoms of facial nerve palsy during diagnostic investigation.

**Diagnoses:**

A fine-needle aspiration cytology showed granulomatous inflammation. The diagnosis of sarcoidosis was eventually established based on the demonstration of the characteristic lambda and panda signs by a Gallium-67 scintigraphy.

**Interventions:**

No specific pharmacologic therapy was initiated.

**Outcomes:**

The patient's symptoms regressed completely over a period of 3 months. Additionally, she remains asymptomatic 2 years later.

**Lessons:**

This case underlines the importance of not initiating corticosteroids in all patients with parotid gland sarcoidosis and suggests that parotidectomy can be avoided in the presence of characteristic for sarcoidosis imaging findings.

## 1 Introduction

Sarcoidosis is a multiorgan disease of unknown origin characterized by the presence of noncaseating granulomas in affected tissues. Corticosteroids remain the mainstay of treatment.^[1]^ Parotitis as a 1st manifestation of sarcoidosis is rare (approximately 1% of cases)^[2]^ and most commonly bilateral (70%).^[3]^ We present a case of unilateral parotid gland sarcoidosis with automatic remission.

## 2 Case presentation

A 28-year-old woman presented with right parotid enlargement. The patient's medical history was remarkable for hypothyroidism and β-thalassemia heterozygosity.

The patient had been diagnosed with left trigeminal neuralgia (TN) 10 weeks earlier. A brain magnetic resonance imaging (MRI) scan showed no pathologic findings. Treatment with pregabalin and a single dose of betamethasone induced successful recession of the symptoms.

Fifteen days before presentation, the patient developed enlargement of the right parotid gland and ipsilateral submandibular area, which was accompanied by a diffuse headache of constant intensity without neurologic symptoms. There were no signs indicative of infection. Moreover, the patient did not complain of a dry mouth or dry eyes.

At presentation the patient was clinically stable and fully active. Clinical examination was unremarkable, except for a 2-cm large, hard, round, irregularly demarcated, painless, and nonmobile mass in the right parotid region. Furthermore, 2 oval-shaped, up to 2 cm large, hard, painless, mobile lymph nodes could be palpated in the right submandibular area.

Microcytic anemia (hemoglobin = 9 g/dL, mean corpuscular volume = 54.9 fL) was the only finding of the initial laboratory workup (baseline hemoglobin 2 months earlier = 10.1 g/dL).

A regional ultrasonography demonstrated an enlarged right parotid gland of lobed margin and inhomogeneous echotexture. Endoparotid lymph nodes were also detected. In addition, 2 enlarged right submandibular lymph nodes (22 and 24 mm) were revealed.

An MRI scan showed diffuse inhomogeneous enlargement of both the superficial and deep parotid lobes and confirmed the presence of submandibular lymphadenopathy. An additional finding was a localized mass in the superficial lobe of the parotid gland (16 mm in diameter) in close contact with the facial nerve. A fine-needle aspiration (FNA) cytology revealed a granulomatous parotitis and lymphadenitis.

Basic laboratory testing for common infectious agents (Epstein–Barr virus, cytomegalovirus, toxoplasma, human immunodeficiency virus, brucella, syphilis, and tuberculosis) was negative. Tests for antinuclear, anti-Ro, and anti-La autoantibodies were negative. A Schirmer test showed normal lacrimation.

A chest X-ray showed right hilar lymphadenopathy that did not obscure the cardiac silhouette. The serum-angiotensin-converting enzyme (SACE) was within normal range (41 IU/L), 24-hour urinary calcium was low (31 mg/24 h) and the patient was vitamin D deficient (25-hydroxyvitamin D = 4.02 ng/mL).

A week later, signs of right facial palsy with concomitant increase in the parotid's size became apparent. Additionally, persistent frontal headache, low grade fever, malaise, lower limb arthralgias and an annular rash in the right knee and gluteal region were added.

**Figure 1:**
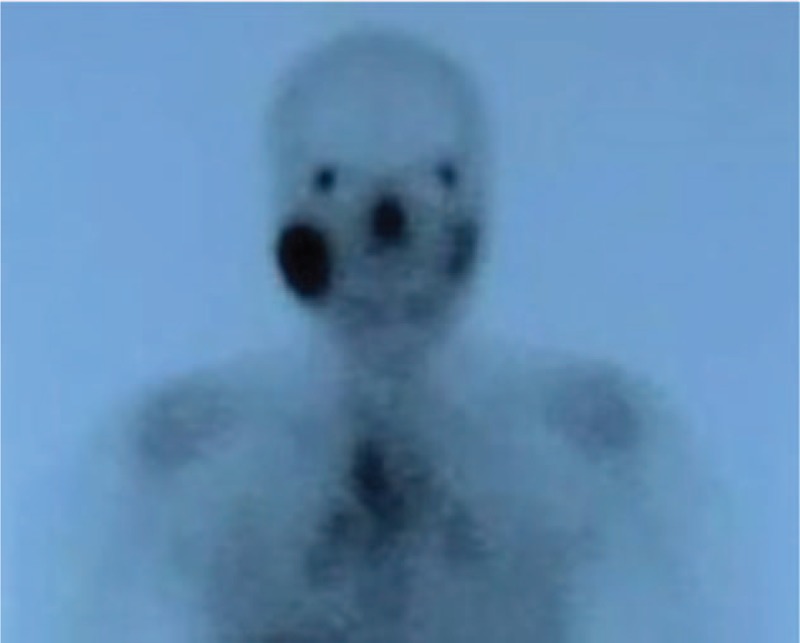
A Gallium-67 scintigraphy demonstrating the typical uptake patterns of panda and lambda signs.

A chest computed tomography (CT) showed enlargement of right paratracheal and hilar nodes bilaterally. In a subsequent Gallium-67 (Ga-67) scintigraphy, the typical uptake patterns of both lambda and panda signs were observed (Fig. 1). Thus, the diagnosis of sarcoidosis was established and the patient was investigated for further organ damage. A new brain MRI did not detect any pathologic findings. A normal electrocardiogram and heart ultrasound excluded cardiac involvement. Finally, a thorough opthalmologic examination ruled out the possibility of uveitis.

No specific pharmacologic treatment was started, because clinical severity was deemed as relatively mild and there were no imaging findings suggestive of central nervous system involvement. Ultimately, over a period of 3 months, lymph node and parotid enlargement regressed completely, the rash resolved, and the patient was free of headache, fever, and symptoms of facial palsy. Interestingly, the hemoglobin level returned to its normal baseline (10.1 g/dL). Two years later, the patient remains free of symptoms.

## 3 Discussion

A malignant cause was initially suspected due to the imaging characteristics of the unilateral parotid enlargement and the concurrent presence of submandibular and hilar lymphadenopathy. Interestingly, it has long been known that metastatic tumors can cause granulomatous inflammation in affected lymph nodes (termed “sarcoid-like” reaction).^[4–6]^

Suspicion for sarcoidosis increased due to the enlargement of hilar lymph nodes that were not contiguous with the cardiac border in the chest X-ray.^[7]^ The typical lymphadenopathy pattern (Garland triad^[8]^), which was revealed in the subsequent chest CT, was also indicative for sarcoidosis. The concurrent demonstration of lambda and panda signs in a Ga-67 scintigraphy is a pathognomonic for sarcoidosis finding. The panda sign is the result of increased Ga-67 uptake in lacrimal and parotid glands bilaterally. This pattern, along with the normal nasopharyngeal radionucleotide marking, resembles the face of a panda. As for the lambda sign, it is produced by the abnormal Ga-67 accumulation in hilar nodes bilaterally and right paratracheal nodes, which is similar to the Greek letter λ.^[9]^

The fact that common biochemical markers of sarcoidosis were not elevated was an additional obstacle in establishing the diagnosis. However, we should take into consideration that SACE levels, which are a marker of overall granulomatous burden, remain within normal range in 15% of patients with sarcoidosis and increase mainly in active pulmonary disease.^[10,11]^ In addition, our patient's vitamin D deficiency could have masked a potential rise in serum and urinary calcium levels.

In summary, panda and lambda signs, granulomatous parotitis, hilar adenopathy, annular rash,^[12]^ and anemia^[13]^ were all suggestive of sarcoidosis. Findings against sarcoidosis were the low calcium and SACE levels, unilateral involvement, no pulmonary disease, and absence of uveitis.^[14]^

Patients with parotid gland sarcoidosis are generally treated with glucocorticoids. Furthermore, in most cases, the diagnosis is established with the demonstration of noncaseating granulomas through parotidectomy. Ungprasert et al studied 345 patients with sarcoidosis. Among those cases, only 7 patients had parotid gland involvement. Oral prednisone was administered in all of them and parotidectomy was conducted in 5 (71.5%).^[2]^

In case reports of patients presenting with parotitis as a 1st manifestation of sarcoidosis, parotidectomy was applied to most of the patients (Table 1).^[15–23]^ We should bear in mind that parotidectomy may be associated with serious complications (e.g., permanent facial nerve damage).^[24]^ Our case indicates that parotid gland surgery should be avoided if there exist strongly suggestive findings of sarcoidosis.

**Table 1:**
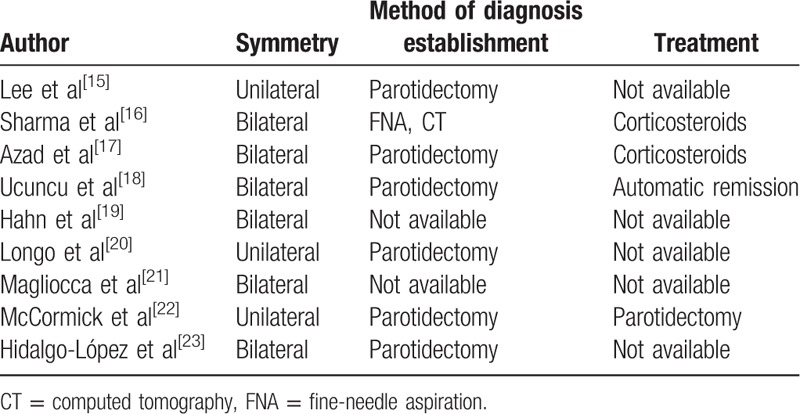
Case reports of parotitis as a 1st manifestation of sarcoidosis: characteristics, diagnostic approach, and treatment strategy.

Contrary to the current consensus in the literature, glucocorticoid therapy was not initiated in our case. Despite lack of specific treatment, her symptoms resolved spontaneously over time. Ucuncu et al also describe the case of a patient who refused treatment with subsequent automatic regression of symptoms.^[18]^ These facts indicate that corticosteroid therapy might not always be necessary in patients who present with parotid gland involvement. Systemic corticosteroid use can cause serious adverse effects and in the above-mentioned subset of patients, who tend to carry a favorable prognosis,^[2]^ the risks might outweigh the benefits.

One could argue that corticosteroid treatment should have been started on the basis of the concomitant facial nerve palsy, which could be indicative of neurosarcoidosis (NS). This possibility could not be excluded, because the diagnosis of NS requires the presence of imaging, cytologic, and/or histopathologic findings indicative of granulomatous inflammation of the nervous system, and this thorough examination was not completed due to the negative MRI.^[25]^ Bell palsy in NS is common (25–50% of patients),^[26]^ but usually self-limited.^[27]^ For this reason, even in confirmed cases of NS, corticosteroid therapy might need to be postponed, especially if the patient's condition does not worsen, if there are no findings indicative of central nervous system involvement and no life-threatening complications. Additionally, we should consider the possibility that the facial nerve palsy could just be the result of nerve compression from the neighboring inflamed parotid gland.

We did not manage to fully explain our patient's headache. Its occurrence and resolution in the same timeframe with symptoms of sarcoidosis indicate that it was disease related. The International Headache Society's diagnostic criteria for “Headache attributed to neurosarcoidosis” require NS evidence,^[28]^ but no cytologic or histologic investigation was conducted. The same applies to our patient's newly diagnosed TN, because the MRI scan conducted at presentation was normal, but this fact does not exclude the possibility of NS. TN is very rare in NS and has only been described in isolated cases.^[29]^

## 4 Conclusion

In conclusion, this case shows that parotid gland sarcoidosis can have a highly atypical presentation and should be included in the differential diagnosis even in unilateral cases. In addition, what makes our case unique is that we managed to avoid parotidectomy by establishing the diagnosis through the demonstration of a pathognomonic imaging finding. Finally, we are among the few authors that suggest that parotid gland sarcoidosis is a self-limited disease which does not require treatment with glucocorticoids.

## Author contributions

**Conceptualization:** Panagiotis T. Diamantopoulos.

**Formal analysis:** Emmanouil Charakopoulos.

**Investigation:** Nora-Athina Viniou, Lydia Diamantopoulou, Maria Gaggadi.

**Methodology:** Panagiotis T. Diamantopoulos.

**Project administration:** Panagiotis T. Diamantopoulos.

**Resources:** Panagiotis T. Diamantopoulos.

**Software:** Emmanouil Charakopoulos.

**Supervision:** Panagiotis T. Diamantopoulos, Nora-Athina Viniou.

**Writing – original draft:** Emmanouil Charakopoulos.

**Writing – review & editing:** Panagiotis T. Diamantopoulos, Nora-Athina Viniou, Lydia Diamantopoulou, Maria Gaggadi.
